# Investing in Public Health Microbiology Laboratories in Western Balkan Countries Enhances Health Security From Communicable Disease Threats in Europe

**DOI:** 10.3389/fpubh.2019.00008

**Published:** 2019-02-04

**Authors:** Agne Bajoriniene, Katrin C. Leitmeyer, Marc J. Struelens, Maarit H. Kokki, Observers and Alternate Observers to the ECDC National Microbiology Focal Points in Western Balkan Countries, Ivana ćirković

**Affiliations:** Author Affiliations: Institute of Microbiology and Immunology, Belgrade, Serbia; National Institute of Public Health, Skopje, the former Yugoslav Republic of Macedonia; University Clinical Center Sarajevo, Bosnia and Herzegovina, Pava Dimitrijević, Public Health Institute of the Republic of Srpska, Banja Luka, Bosnia and Herzegovina; Tirana University Hospital Centre, Tirana, Albania; National Institute of Public Health and University of Pristina, Pristina, Kosovo; National Institute of Public Health and University of Pristina, Pristina, Kosovo; Institute of Public Health of Serbia Dr. Milan Jovanovic Batut, Belgrade, Serbia; Institute of Microbiology and Parasitology, Skopje, the former Yugoslav Republic of Macedonia; National Institute of Public Health, Podgorica, Montenegro; National Institute of Public Health, Tirana, Abania.; ^1^International Relations Section, European Centre for Disease Prevention and Control, Stockholm, Sweden; ^2^Microbiology Coordination Section, Office of the Chief Scientist, European Centre for Disease Prevention and Control, Stockholm, Sweden

**Keywords:** Western Balkan countries, public health microbiology, external quality assessment, molecular diagnostics, antimicrobial susceptibility testing, communicable disease surveillance, cross-border threats, international relations

## Abstract

The European Centre for Disease Prevention and Control (ECDC), under the EU enlargement policy, has supported national efforts of Western Balkan countries to strengthen their communicable disease prevention and control systems. The new EU strategy “A credible enlargement perspective for and enhanced EU engagement with the Western Balkans” advocates transformation processes that will build the foundation of EU-oriented national reforms. Well-functioning public health microbiology laboratories are key for early detection and control of infectious diseases, and thus maintaining and enhancing health security in Europe. In order to help Western Balkan countries to improve their national capacities, ECDC facilitated needs assessments and identified key areas for advancement toward effective public health microbiology systems. Countries identified gaps in their laboratory data reporting and exchange systems. Harmonized and effective procedures for handling of highly contagious agents and cross-border transportation of biological samples were often lacking, as well as the systematic use of diagnostic testing at the primary care level or referral of patients, in particular for detection of antimicrobial resistance. There is a clear need to address the financial investment required for sustaining sufficient numbers of skilled laboratory workforce, laboratory supplies, and the development of new methods and techniques, including investment in emerging laboratory technologies, such as molecular typing by whole genome sequencing. This article highlights the key areas for investing in public health microbiology laboratories in Western Balkan countries needed to strengthen health security in Europe.

## Introduction

Europe's responses to cross-border health threats from communicable diseases are dependent on strong and well-functioning health systems in the EU and in its neighbors. Investing in neighboring countries' epidemiological and laboratory capacities is key for the security of both EU citizens and their neighbors against the threat of communicable diseases. In this regard, ECDC under the EU enlargement policy contributed to national efforts of EU candidate and potential candidate countries to strengthen their communicable disease prevention and control capacities.

Strong capacity of public health microbiology laboratory systems is crucial in providing timely and reliable information on pathogen detection and characterization needed for effective infectious diseases prevention and control, especially in response to epidemic threats.

During the 10 years of technical cooperation on communicable disease prevention and control, ECDC has supported national authorities in EU candidate and potential candidate countries to improve the capacities of microbiology laboratory systems to fulfill requirements set out by *EU acquis* on cross-border health threats. Analysis of the current situation of microbiology laboratories supporting public health in the countries suggests that system capacities and their services to support communicable disease surveillance and response as required by the *EU acquis* are limited. In addition, limited availability of clinical guidelines and laboratory testing algorithms for aetiological diagnosis of common infections arguably hampers efficient treatment of patients in many of these countries.

The new EU strategy for A credible enlargement perspective for and enhanced EU engagement with the Western Balkans confirms the European future of the region based on common values and application of EU rules and standards not only by law, but also in practice (1). The Strategy sets out six new flagship initiatives to support the transformation process in the Western Balkans. Through these six initiatives, the EU encourages Western Balkan countries to reinforce their sustained efforts and irreversible EU-oriented reforms and confirms a credible enlargement perspective, as part of a larger strategy to strengthen the Union by 2025. The dynamics of moving forward on their respective EU paths for all Western Balkans is based on their own merits and at their own speed depending on the concrete results achieved.

The Strategy spells out the need to develop the digital society in the Western Balkans, including the support to eHealth services. Furthermore, the transformation process will be reinforced in socio-economic development, including investment in health to support social inclusion. In-depth analysis of information gathered by ECDC about Western Balkan countries' capacities in the area of communicable disease prevention and control suggests that despite a number of positive achievements in national microbiology laboratory service capabilities, there is an urgent need for these countries to further improve their public health microbiology systems before they join the EU.

## ECDC's Support to Laboratory Capacities in Western Balkan Countries

In line with the current ECDC International Relations Policy 2020 (2), EU pre-accession countries (Albania, Bosnia and Herzegovina, Kosovo^1^, Montenegro, Serbia, the former Yugoslav Republic of Macedonia, and Turkey) have been the main beneficiaries of ECDC technical assistance for non-EU/EEA countries over the last 10 years. With financial support from the European Commission under Instrument of Pre-accession Assistance (IPA) (3), ECDC has gradually supported integration of these countries into ECDC activities, including support to public health microbiology systems. Upon request from the European Commission, ECDC has also been assessing country capacities in the area of communicable disease prevention and control. ECDC reviewed countries' compliance and implementation of EU legislation and made recommendations on reforms needed to meet essential public health system requirements as part of the accession process. In this regard, one of the six assessed areas was national systems of public health microbiology laboratories.

In 2013, an Observer role was established to the ECDC National Microbiology Focal Points (NMFP) forum for Western Balkan countries and Turkey representing national public health institutes, to increase awareness about the importance of public health microbiology in national communicable disease surveillance and control, thereby contributing to the preparation of these countries to participate in ECDC work. Through this initiative, ECDC supports implementation of EU acquis through exchange of best practices and EU standards for effective laboratory response to public health events and enhanced public health microbiology.

The evaluation (performed in 2014) of the pilot initiative and implementation of related country action plans in six Western Balkan countries suggested that establishing the Observer role to NMFP facilitated national efforts to improve antimicrobial resistance surveillance. A majority of countries reported that being part of the ECDC microbiology expert network helped them to prepare for implementation of EUCAST clinical breakpoints for standardized antimicrobial susceptibility testing and to become familiar with EU-level standards as per the EU protocol for harmonized monitoring of antimicrobial resistance in human *Salmonella* and *Campylobacter* isolates to enable One Health collaboration between colleagues from the human health and veterinary medicine sector (4). This observer role to the network also facilitated participation in WHO/Europe coordinated Central Asian and Eastern European Surveillance of Antimicrobial Resistance (CAESAR) network (5), and assisted in the establishment of inter-sectorial committees on antimicrobial resistance at the national level.

The assessments of communicable diseases surveillance and control systems in four EU candidate countries (Montenegro, Serbia, the former Yugoslav Republic of Macedonia, Albania) suggest a common need to define and further strengthen public health microbiology laboratory systems to attain an adequate level of capability to provide timely and reliable information on pathogen detection and characterization for effective infectious disease treatment, prevention, alert, and control. The Regional Seminar on Communicable Diseases Surveillance in Budva, Montenegro (2016) recognized that countries in the region need to improve the integration of laboratory services into their surveillance system and develop or improve national laboratory networks.

ECDC supported participation of EU candidate and potential candidate countries in some ECDC-coordinated external quality assessment (EQA) schemes. These EQAs covered methods from pathogen detection and identification, molecular typing, to antimicrobial susceptibility testing (AST). Participation in these EQA exercises is still limited and suggests that there are gaps in capacities to perform molecular typing for certain pathogens (Table 1).

**Table 1:** Western Balkan countries participation in ECDC-supported laboratory external quality assessment schemes offered to EU pre-accession countries, by target pathogen, testing area, and year.

**Pathogen and testing area**	**Number of countries participating by year**					
	**2012**	**2013**	**2014**	**2015**	**2016**	**2017**
*Salmonella*, AST	–	–	–	5	5	5
*Campylobacter*, AST	–	–	–	4	4	5
*Salmonella spp.*, PFGE and MLVA molecular typing	1	1	2	2	^*^	2
*STEC/VTEC*, PFGE, serotyping, virulence gene detection, and/or molecular typing	1	1	1	2	^*^	1
*Listeria monocytogenes*, serotyping, and molecular typing	^*^	–	1	2	^*^	1
*Mycobacteria tuberculosis*, culture, NAAT, AST	^*^	–	2	4	5	6

* *EQA was not offered*.

A regional ECDC multi-country workshop on microbiology laboratory systems supporting public health in EU enlargement countries was organized in 2017 to reflect on progress achieved in reviewing and re-organizing their national laboratory networks and to identify gaps at the national level related to the reference laboratory functions. In addition countries' delegates discussed the feasibility of sharing expertise across countries and possible mechanisms to enable certain laboratories to serve as reference laboratories for other countries in the region. In preparation for the ECDC Multi-country workshop on microbiology laboratory systems supporting public health, in June 2017, in Ohrid (6), the six Western Balkan countries (Albania, Bosnia Herzegovina^2^, the former Yugoslav Republic of Macedonia, Montenegro, Kosovo, and Serbia) completed a pre-workshop questionnaire using 2016 data. This questionnaire was sent to officially nominated Observers to the ECDC NMFP who coordinated the collection of data at national level.

## Capacities of Public Health Microbiology Laboratory Systems of Western Balkan Countries as Per *EU Acquis* in 2016

With respect to country capacities to confirm cases by primary and/or reference laboratory testing for 53 notifiable communicable diseases as per *EU acquis* (7) (as per EU surveillance case definition) (8), the capacity in the Western Balkan countries ranged between 70 (37 out of 53 diseases) and 85% (45 out of 53 diseases). No country reported having full diagnostic capacity for variant Creutzfeldt-Jacob disease, and only a few countries reported full capacity for rare and emerging diseases, such as smallpox, SARS, and yellow fever. For botulism, plague, poliomyelitis, rabies, tetanus, and yersiniosis the diagnostic capacity was limited. An overview of capacities by pathogen is displayed in Table 2.

**Table 2:** Western Balkan countries' capacities to confirm cases by primary and/or reference laboratory for the following notifiable 53 communicable diseases as per *EU acquis*.

**Disease under EU surveillance**	**Number of countries**
ACQUIRED IMMUNODEFICIENCY SYNDROME (AIDS) AND HUMAN IMMUNODEFICIENCY VIRUS (HIV) INFECTION	6
AVIAN INFLUENZA A/H5 OR A/H5N1 IN HUMANS	
BRUCELLOSIS (*Brucella* spp.)	
CAMPYLOBACTERIOSIS (*Campylobacter* spp.)	
CHLAMYDIAL INFECTION (*Chlamydia trachomatis*), INCLUDING LYMPHOGRANULOMA VENEREUM (LGV)	
CHOLERA (*Vibrio cholerae*)	
DIPHTHERIA (*Corynebacterium diphtheriae, Corynebacterium ulcerans* and *Corynebacterium pseudotuberculosis*)	
ECHINOCOCCOSIS (*Echinococcus* spp.)	
GIARDIASIS (*Giardia lamblia*)	
GONORRHEA (*Neisseria gonorrhoeae*)	
HAEMOPHILUS INFLUENZAE, INVASIVE DISEASE (*Haemophilus influenzae*)	
HEPATITIS A (Hepatitis A virus)	
HEPATITIS B (Hepatitis B virus)	
HEPATITIS C (Hepatitis C virus)	
INFLUENZA (Influenza virus)	
INFLUENZA A(H1N1)	
LEGIONNAIRES' DISEASE (*Legionella* spp.)	
LEPTOSPIROSIS (*Leptospira* spp.)	
LISTERIOSIS (*Listeria monocytogenes*)	
MALARIA (*Plasmodium* spp.)	
MEASLES (Measles virus)	
MENINGOCCOCAL DISEASE, INVASIVE (*Neisseria meningitidis*)	
MUMPS (Mumps virus)	
PERTUSSIS (*Bordetella pertussis*)	
PNEUMOCOCCAL INVASIVE DISEASE(S) (*Streptococcus pneumoniae*)	
Q FEVER (*Coxiella burnetii*)	
RUBELLA (Rubella virus)	
RUBELLA, CONGENITAL (including Congenital Rubella Syndrome)	
SALMONELLOSIS (*Salmonella* spp. other than *Salmonella* Typhi and *Salmonella* Paratyphi)	
SHIGELLOSIS (*Shigella* spp.)	
SYPHILIS (*Treponema pallidum*)	
SYPHILIS, CONGENITAL AND NEONATAL (*Treponema pallidum*)	
TOXOPLASMOSIS, CONGENITAL (*Toxoplasma gondii*)	
TUBERCULOSIS (*Mycobacterium tuberculosis* complex)	
TYPHOID/PARATYPHOID FEVER (*Salmonella* Typhi/Paratyphi)	
VIRAL HAEMORRHAGIC FEVERS (VHF)	
WEST NILE FEVER (West Nile virus infection, WNV)	
ANTHRAX (*Bacillus anthracis*)	5
CRYPTOSPORIDIOSIS (*Cryptosporidium* spp.)	
TICK-BORNE ENCEPHALITIS (TBE virus)	
YERSINIOSIS (*Yersinia enterocolitica, Yersinia pseudotuberculosis*)	
SEVERE ACUTE RESPIRATORY SYNDROME—SARS (SARS-coronavirus, SARS-CoV)	4
TRICHINELLOSIS (*Trichinella* spp.)	
TULARAEMIA (*Francisella tularensis*)	
TETANUS (*Clostridium tetani*)	3
BOTULISM (*Clostridium botulinum*)	2
POLIOMYELITIS (Polio virus)	
RABIES (Lyssa virus)	
SHIGA TOXIN/VEROCYTO-TOXIN PRODUCING ESCHERICHIA COLI INFECTION (STEC/VTEC)	
YELLOW FEVER (Yellow fever virus)	
CREUTZFELDT-JAKOB DISEASE, VARIANT (vCJD)	1
PLAGUE (*Yersinia pestis*)	
SMALLPOX (Variola virus)	0

Countries identified several key issues in the organizational set up and structures of national laboratory systems supporting public health that need to be addressed. First, national regulations and nominations of reference microbiology laboratories are often missing or ambiguous; re-assessment of the situation for re-nomination is problematic in a majority of countries. Second, resources for laboratory activity are often ineffectively allocated to e.g., screening programmes without scientific basis and tangible impact (and lacking technical collaboration with other sectors). Third, in some countries, there is no complete inventory nor a clear overview of all microbiology laboratories; only half of the countries have a national database of all available microbiology laboratories operating in the public and private sectors. Four out of six countries reported that they perform a national assessment of capacities, space, equipment, and staff. None of the countries has established an electronic laboratory data reporting system from microbiology laboratories to the public health central level. All countries reported having established cross-sectoral information exchange including exchange between laboratory and epidemiology services.

All six countries have the capacity to identify methicillin-resistant *Staphylococcus aureus* (MRSA) isolates in accordance with EUCAST/*Staphylococcus aureus* reference laboratory network guidance. This reflects the efforts of a wider implementation of the recommendations by the European Committee on Antimicrobial Susceptibility testing (EUCAST) (9) in collaboration with national susceptibility committees, which most of the Western Balkan countries have established.

In terms of capacity on national level to perform molecular typing, half of the countries have the capacity for characterizing multidrug resistant *Mycobacterium tuberculosis*, whereas only one country was able to genotype *Salmonella enterica* and *Listeria monocytogenes*. None of the countries has the capability to perform molecular typing for Shiga-toxin producing *E.coli*.

Concerning laboratory capacity for the detection of common diseases, all countries lack capacity for diagnostics of some diseases/syndromes important for patient management and safety, and monitoring of public health programs, particularly on the regional and local level. All countries reported that laboratories serving physicians at the primary care level have insufficient technical equipment or reagents and that adequate diagnostic tests are therefore not available. In addition, in all but one country doctors do not systematically test and/or refer patients for diagnostic microbiology testing. In some countries, standard algorithms for performing laboratory testing of samples in patients presenting with common diseases/syndromes are either not available or not followed at clinical care level. All countries except one have developed algorithms for sample referral for meningitis, however only half of the countries have algorithms for sample referral for cases of diarrhea. Four countries indicated that insufficient levels of trained laboratory personnel in clinical laboratories also hamper effective use of microbiology diagnostic tests by physicians at primary level.

Moreover, in a number of countries, business continuity is a problem for diagnostic services because of complex procurement processes or insufficient funding, and lack of appropriately trained staff. In particular, there is a lack of support for clinical and reference laboratories for enteric disease/diarrhea diagnostics, which jeopardizes recognition of outbreaks with foodborne pathogens.

In addition, there are capacity gaps in antimicrobial resistance testing for patient management and antibiotic policy guidance in the region. Only half of the countries indicated having a national policy for cross-sectoral and coordinated monitoring of antimicrobial resistance in human and animal bacterial isolates of public health relevance.

All countries except one had laboratory specialists systematically involved in preparedness planning and outbreak investigations at all levels. Even if many national reference laboratories and some clinical microbiology laboratories participate in external quality assessments (EQA), in general the national implementation of EQA is either patchy or missing in all countries. None of the countries has external quality assessment systems covering all the microbiology laboratories in the country.

## Perceived Needs of Countries to MODERNIZE Microbiology Laboratory Processes and Structures for Support of Communicable Disease Surveillance and Control

Apart from financial investment and adequate numbers of laboratory personnel, Western Balkan countries reported the need for support in managing the technology transition to molecular surveillance and cluster detection methods as priority areas for action. Half of the countries also identified the need to facilitate the setting-up of effective electronic data reporting and exchange systems for microbiology information.

All countries expressed the need to develop new and innovative microbiology methods and techniques, including next generation sequencing (NGS)/whole genome sequencing (WGS), and training of specialists to interpret microbial NGS/WGS results when samples are sent abroad for analysis. In addition, formalizing agreements and working arrangements with other institutions for technical collaboration, including addressing legal and practical barriers, was seen as an important step to increase capabilities for microbiology laboratory services for effective public health.

The Western Balkan region reported significant gaps in harmonized and effective procedures and logistical arrangements on cross-border transportation and handling of specimens of highly contagious agents. This is possibly due to a lack of legal basis/regulations enabling efficient sample transferral and intergovernmental collaboration.

All Western Balkan countries acknowledged that their persistent financial constraints, if not addressed sufficiently, will ultimately lead to decreased numbers of laboratory workforce, shortage of qualified laboratory personnel, and insufficient laboratory materials and reagents. This is likely to jeopardize effective microbiology laboratory capacities to support clinical and public health response in the region and beyond.

## Approaches to Address Microbiology Laboratory System Gaps of Western Balkan Countries on Their way to Meet the Requirements of EU Membership

This overview of the 2016 capacities of public health microbiology laboratory systems in six Western Balkan countries identifies several priorities for tackling the regional gaps.

To address the sustainability of financial resources for microbiology laboratory services supporting public health there is an opportunity for innovative approaches such as re-directing resources through structural re-organization, sharing of services between sectors, and cross-border collaboration.

To guarantee that primary care physicians effectively use microbiological diagnostic tests, countries need to address key issues of availability of diagnostic and screening tests and raise physicians' awareness and understanding on the benefits of testing to the patient and to the public health. In addition, key areas/programmes (including enteric disease/diarrhea diagnostics, AMR testing for patient management, and antibiotic policy guidance) require high quality support both from clinical and reference laboratories.

There is a need to clarify and better define licensing-accreditation processes and to ensure that the quality of laboratory services not only requires good coverage of EQAs, but also access to up to date technical infrastructure and well trained personnel.

To increase capabilities for microbiology laboratory services and to develop them further, EU pre-accession countries should ensure that formal agreements for collaboration are in place within and outside the countries. They should also consider application of new and emerging microbiology laboratory methods and techniques.

To remedy the problems related to transportation of specimens and handling of samples a regulatory framework for sample transfers is needed (within and outside the country), including e.g., the organization of the transportation process, handling of specimens, and intergovernmental collaboration.

To implement the policies/strategies on microbiology laboratory systems including the necessary technical advancements it is essential to build professional and institutional partnerships and seek functional partners e.g., from EU Member States, while ensuring the national commitment for sustainability.

Annual monitoring of laboratory capabilities based on the standardized ECDC EULabCap indicator tool (10) customized for EU pre-accession countries (including individualized country reports with benchmarking against EU standards) could help countries to promote necessary changes in national public health microbiology systems and initiate their implementation, as well as to advocate for sustainable financing mechanisms to ensure the delivery of reforms. In this way, countries will be equipped to address areas that need political attention.

## Outlook, the way Forward

The EU has reaffirmed a credible enlargement perspective for the Western Balkans (11). The EU's enlargement policy, currently in transition into a broader strategy to strengthen the Union by 2025, was and continues to be an investment in the EU's security, economic growth, and influence. Reinforced EU's investment under the updated flagship initiatives to support transformation of Western Balkans in their perspective toward EU enlargement, opens an opportunity for assistance to deliver reforms needed to implement *EU acquis*, including addressing health security threats and developing digital society. In this regard, based on the in-depth analysis of information on countries' capacities in communicable disease prevention and control, there is an urgent need for Western Balkan countries to improve their public health microbiology systems before they join the EU to guarantee optimal outbreak detection, control measures, and patient management.

Bringing together advanced technologies in laboratory diagnostics (e.g., NGS- and/or WGS-based typing) and epidemiology raises the possibility of a digital pathogen surveillance system. In the context of the One Health approach, in which human, animal, and environmental health are considered together, such a system could have a potential to improve public health, e.g., in settings lacking robust laboratory capacity (12), such as in countries of the Western Balkan region. Thus, there is also a clear case to be made to integrate improvement of laboratory capacities and capabilities for effective pathogen detection, characterization and control into regional investment to boost One Health approach against AMR in Western Balkan countries.

Building resilient and responsive public health systems in countries of EU's neighborhood entails more than just enhancing technical capacities of epidemiology and public health microbiology systems. It is also about engagement, cooperation and partnerships for future actions. Bilateral and multilateral cooperation in the region of all EU pre-accession countries, coordinated activities with WHO Regional Office for Europe, technical cooperation between competent institutions of EU Member States and Western Balkan countries, and using available expertise in the adjoining countries is imperative for the enhancement of health security from communicable disease threats in Europe.

## Author Contributions

AB and KL: data collation, analysis, and writing the draft; MS and MK: reviewing and revising the draft.

### Conflict of Interest Statement

The authors declare that the research was conducted in the absence of any commercial or financial relationships that could be construed as a potential conflict of interest.

## Footnotes

^1^This designation is without prejudice to positions on status, and is in line with UNSCR 1244/1999 and the ICJ Opinion on the Kosovo declaration of independence.

^2^Disclaimer: some responses from Bosnia Herzegovina were only made for the Federation of Bosnia but not for the Republic of Srpska.
